# LL-37 alone and in combination with IL17A enhances proinflammatory cytokine expression in parallel with hyaluronan metabolism in human synovial sarcoma cell line SW982—A step toward understanding the development of inflammatory arthritis

**DOI:** 10.1371/journal.pone.0218736

**Published:** 2019-07-01

**Authors:** Chakkrapong Kuensaen, Siriwadee Chomdej, Patiwat Kongdang, Nutnicha Sirikaew, Rungnaree Jaitham, Supitcha Thonghoi, Siriwan Ongchai

**Affiliations:** 1 Department of Biology, Faculty of Science, Chiang Mai University, Chiang Mai, Thailand; 2 Thailand Excellence Center for Tissue Engineering and Stem Cells, Department of Biochemistry, Faculty of Medicine, Chiang Mai University, Chiang Mai, Thailand; Univerzitet u Beogradu, SERBIA

## Abstract

LL-37 is the only human cathelicidin-family host defense peptide and has been reported to interact with invading pathogens causing inflammation at various body sites. Recent studies showed high levels of LL-37 in the synovial-lining membrane of patients with rheumatoid arthritis, a common type of inflammatory arthritis. The present study aims to investigate the role of LL-37 on mechanisms associated with pathogenesis of inflammatory arthritis. The effects of LL-37 on the expression of proinflammatory cytokines, hyaluronan (HA) metabolism-related genes, cell death-related pathways, and cell invasion were investigated in SW982, a human synovial sarcoma cell line. Time-course measurements of proinflammatory cytokines and mediators showed that LL-37 significantly induced *IL6* and *IL17A* mRNA levels at early time points (3–6 hr). HA-metabolism-related genes (i.e., HA synthase 2 (*HAS2*), *HAS3*, hyaluronidase 1 *(HYAL1*), *HYAL2*, and *CD44*) were co-expressed in parallel. In combination, LL-37 and IL17A significantly enhanced *PTGS2*, *TNF*, and *HAS3* gene expression concomitantly with the elevation of their respective products, PGE2, TNF, and HA. Cell invasion rates and *FN1* gene expression were also significantly enhanced. However, LL-37 alone or combined with IL17A did not affect cell mortality or cell cycle. Treatment of SW982 cells with both LL-37 and IL17A significantly enhanced IKK and p65 phosphorylation. These findings suggest that the chronic production of a high level of LL-37 may synchronize with its downstream proinflammatory cytokines, especially IL17A, contributing to the co-operative enhancement of pathogenesis mechanisms of inflammatory arthritis, such as high production of proinflammatory cytokines and mediators together with the activation of HA-metabolism-associated genes and cell invasion.

## Introduction

Inflammatory arthritis is a group of diseases characterized by inflammation of the joints or surrounding tissues. Rheumatoid arthritis (RA) is one of the most common inflammatory arthritis diseases. It is an autoimmune disease characterized by the chronic inflammation of the synovial membrane. It causes joint swelling, abnormal growth of the synovial tissue, and progressive invasion of synovial fibroblasts into the articular cartilage and the underlying bone, leading to joint destruction [1]. Potential causes of inflammatory arthritis and RA are thought to involve both genetic and environmental factors [2, 3], and its pathogenesis involves the high production of proinflammatory cytokines, such as tumor narcosis factor (TNF), interleukin 1 beta (IL1B), interleukin 6 (IL6), and interleukin 17A (IL17A), inducing autoimmune activity, thereby inflaming the synovial membrane [4]. Inflamed synoviocytes then divide uncontrollably, due to the impairment of programed cell death pathways, including apoptosis, necroptosis, and autophagy [5].

Hyaluronan (HA) polymer accumulation and turnover are also associated with inflammation. HA regulates the expression of inflammatory genes in a manner dependent on HA molecular weight, as evidenced by a growing number of reports [6], and several studies have indicated its close involvement in inflammatory arthritis development [7, 8]. High molecular weight (HMW) HA, produced by hyaluronan synthase 1 (HAS1) and HAS2, exerts anti-angiogenic, immunosuppressive, and anti-inflammatory properties [9, 10], whereas low-molecular-weight (LMW) HA, which is synthesized by HAS3, is usually secreted from cells both under physiological and pathological conditions [11]. This LMW-HA functions oppositely to HMW-HA, exerting proinflammatory, angiogenic, and immunostimulatory roles [12]. HA degradation occurs mainly through hyaluronidase (HYAL) enzymes, especially HYAL1 and HYAL2 [13, 14], and there is increasing evidence that HA degradation products activate inflammation in injury and disease [15]. The major cell-surface receptors for HA are CD44 and TLR4, which are a widely distributed transmembrane glycoprotein involved in a wide variety of biological processes, including cell adhesion and migration, as well as inflammation and cancer [16]. HA interaction with CD44 triggers PI3K/PDK1/Akt and ERK1/2 activity. It also helps cell adhesion, cell migration, and cell infiltration. However, this migration was reported to be specific for the interaction with HMWHA [17]. On the other hand, TLR4 is known for its ability to selectively activate NF-κB proteins and induce proinflammatory cytokines. This selectivity depends strongly on the size of HA and TLR clustering pattern on cell membrane [15].

Cathelicidin is a family of host defense peptides found in vertebrates. In humans, there is only one cathelicidin, designated as LL-37 [18]. Although LL-37 has been known for its anti-microbial activities [19], its immunomodulatory functions have gained considerable interest over the past decade. LL-37 has been reported to be involved in both pro- and anti-inflammatory pathways, in different environments [20]. For example, in human gingival fibroblasts, LL-37 was found to enhance prostaglandin-endoperoxide synthase 2 (PTGS2 or COX2) expression and prostaglandin E2 (PGE2) production via ERK and c-Jun-N-terminal kinase [21], whereas in human skin, LL-37 was shown to promote proliferation, invasion, and chemokine production [22, 23]. RA patients present elevated levels of LL-37 in the synovial tissues, suggesting its involvement in the development of inflammatory arthritis [20, 24].

To date, the mechanisms underlying the involvement of LL-37 in inflammatory arthritis and RA pathogenesis are poorly understood, especially in combination with other proinflammatory cytokines. The present study aimed to investigate the effects of LL-37 alone and in combination with IL17A, on the mechanisms related to inflammatory arthritis pathogenesis in the human synovial sarcoma cell line, SW982. The expression of proinflammatory cytokines and HA-metabolism-associated genes were examined, as well as cell death pathways and cell invasion.

## Materials and methods

### SW982 cell culture

A human synovial sarcoma cell line (SW982) was purchased from the American Type Culture Collection (Manassas, VA, USA) and authenticated by the European Collection of Authenticated Cell Culture, in May 2017. The cells were cultured in Leibovitz’s L-15 medium (Gibco, Thermo Fisher Scientific, Pittsburgh, PA, USA) supplemented with 100 U/mL penicillin, 10 μg/mL streptomycin, and 10% fetal bovine serum (FBS, Thermo Fisher Scientific) in a humidified atmosphere. The cells were incubated in serum-free medium for 24 hr prior to LL-37 (6.25–50 ng/mL, ProSpec) and/or recombinant IL17A (5 and 10 ng/mL, ProSpec) treatment, for 3–72 hr depending on the experiments. The cells were then collected for gene expression analysis, flow cytometric assay, caspase-8 activity assay, cell cycle assay, and western blot analysis. Culture media were collected for measurements of HA, TNF, and PGE2 levels.

### Determination of hyaluronan, TNF, and PGE2 levels by ELISA

The levels of HA were measured from 3-day culture supernatant using a competitive inhibition ELISA, according to the protocol by Viriyakhasem et al. [25]. Briefly, samples or standard HA (Healon) at various concentrations (19–10,000 ng/mL) in PBS pH 7.4, were added to 1.5 mL plastic tubes containing biotinylated HABPs. The tubes were incubated at room temperature for 1 hr and, then, samples were added to a 96-well microplate, which was pre-coated with umbilical cord HA, and blocked with 1% BSA. The plate was then incubated at room temperature for 1 hr. The wells were subsequently washed and 100 μL of peroxidase-conjugated anti-biotin antibody was added. The plate was incubated at room temperature for another hour. The reaction was stopped after 10 min with sulfuric acid and the absorbance was determined using a microplate reader at 492/690 nm. The concentration of HA in samples was calculated by referring to a standard curve. TNF and PGE2 levels were also measured from 3-day culture supernatant, using commercial ELISA kits (Bender Med Systems and Elabsciences, respectively), according to the manufacturer’s instructions.

### Measuring gene expression by quantitative real-time PCR

The cells were extracted for total RNA using the RNAspin Mini (GE Healthcare UK Ltd, Buckinghamshire, UK) following manufacturer’s instructions. The total RNA was reverse-transcribed into cDNA using the Tetro cDNA Synthesis Kit (Bioline, MA, USA). The cDNA was amplified for 35 cycles using specific primers (S1 Table). Quantitative data were calculated using the 2^-ΔΔCT^ method and normalized to *GAPDH* expression.

### Invasion assays

Transwell chambers (8 μm, 24-well format; Corning Co., USA) precoated with 15 μg/μL of Geltrex Matrix (Thermo Fisher Scientific) were used, inserted into 24-well cell culture plates. Cells (3 × 10^4^ in 0.2 mL of serum-free medium) were added to the upper chamber, while 0.6 mL of L-15 medium containing 20% FBS were added to the lower chamber. The cells were cultured for 24 hr, and those invading through the inserts were fixed in methanol for 20 min, stained with crystal violet, and photographed in three random fields under a microscope (Olympus BX3, Japan) at a 40x magnification. Then, Image J (1.52b, National Institutes of Health, USA) was used to measure the stained areas.

### Flow cytometric analysis for caspase-3/7 activity

The cell samples were evaluated for apoptotic cells using the Muse Caspase-3/7 Kit according to the manufacturer’s instructions. Briefly, SW982 cells were seeded into 6-well plates (1.5 x 10^5^ cells/well), starved with serum-free medium for 24 hr, and treated with LL-37, IL17A, or DMSO for 48 hr. Then, the cells were collected with trypsin, washed by phosphate buffer saline (PBS), and incubated with Muse Caspase-3/7 and 7-AAD reagent, prior to being run on the Muse Cell Analyzer.

### Caspase-8 activity assay

Caspase-8 activity was measured using the Caspase-8 Colorimetric Activity Assay Kit’s (Chemicon International) protocol. Briefly, SW982 cells were plated into 6-well plates, starved with serum-free medium for 24 hr, treated with LL-37, IL17A, or DMSO for 48 hr. Then, the cell lysates were collected, were assayed for protein concentration using the protein assay (Bio-Rad), were incubated with assay mixture, and were measured for optical density at 405 nm with a micro-plate reader, together with standards.

### Cell cycle assay for cell proliferation

SW982 cells were plated into 6-well plates, starved in serum-free medium for 24 hr, treated with LL-37, IL17A, or 5% FBS for 48 hr, and collected by trypsinization. Then, the cells were centrifuged at 500 g for 5 min, washed with 1000 μL of PBS twice, fixed with 500 μL of cold 70% ethanol for 30 min, washed with PBS, incubated with 200 μL of 100 μg/mL of ribonuclease A and 50 μg/mL of propidium iodide (PI) on ice for 30 min, washed with PBS and analyzed by BD FACS Canto II (BD Biosciences).

### Western blot analysis

The SW982 cell line was treated with LL-37 (ProSpec) 12.5 ng/mL and/or IL17A (ProSpec) 10 ng/mL, or BAY11-7082 (Calbiochem) 20 μM, for exactly 10 min. The cell lysates were collected into the radioimmunoprecipitation assay buffer and the samples were subjected to SDS-PAGE under a reducing condition. The proteins were electrotransferred onto a nitrocellulose membrane. The blots were detected with antibodies (Cell Signaling Technology) specific for phospho- and total IKK, IκB, and p65 in the NF-κB signaling pathway. Then, the bands were developed and detected using horse-peroxidase-conjugated antibodies and the ECL chemiluminescence detection kit. The total form of each molecule was determined from the same membranes that were used to quantify the phosphorylated forms by stripping with stripping buffer (Thermo Scientific). The intensity level of each band was analyzed with Image J (1.52b, National Institutes of Health, USA) and normalized by the intensity of β-actin. The intensity of phosphorylated forms was then re-normalized by the intensity of their total forms.

### Statistical analysis

RStudio version 1.0.44 with R version 3.3.2 and Microsoft Excel for Mac version 16.20 were used to perform all statistical analyzes. The results are presented as the mean ± SD of two or three independent replicates. Assuming the normal distribution of data, statistically significant values were compared using one-way analysis of variance, followed by Tukey’s honestly significant difference (HSD) post-hoc test. A level of *P* < 0.05 was considered statistically significant.

## Results

**Fig 1:**
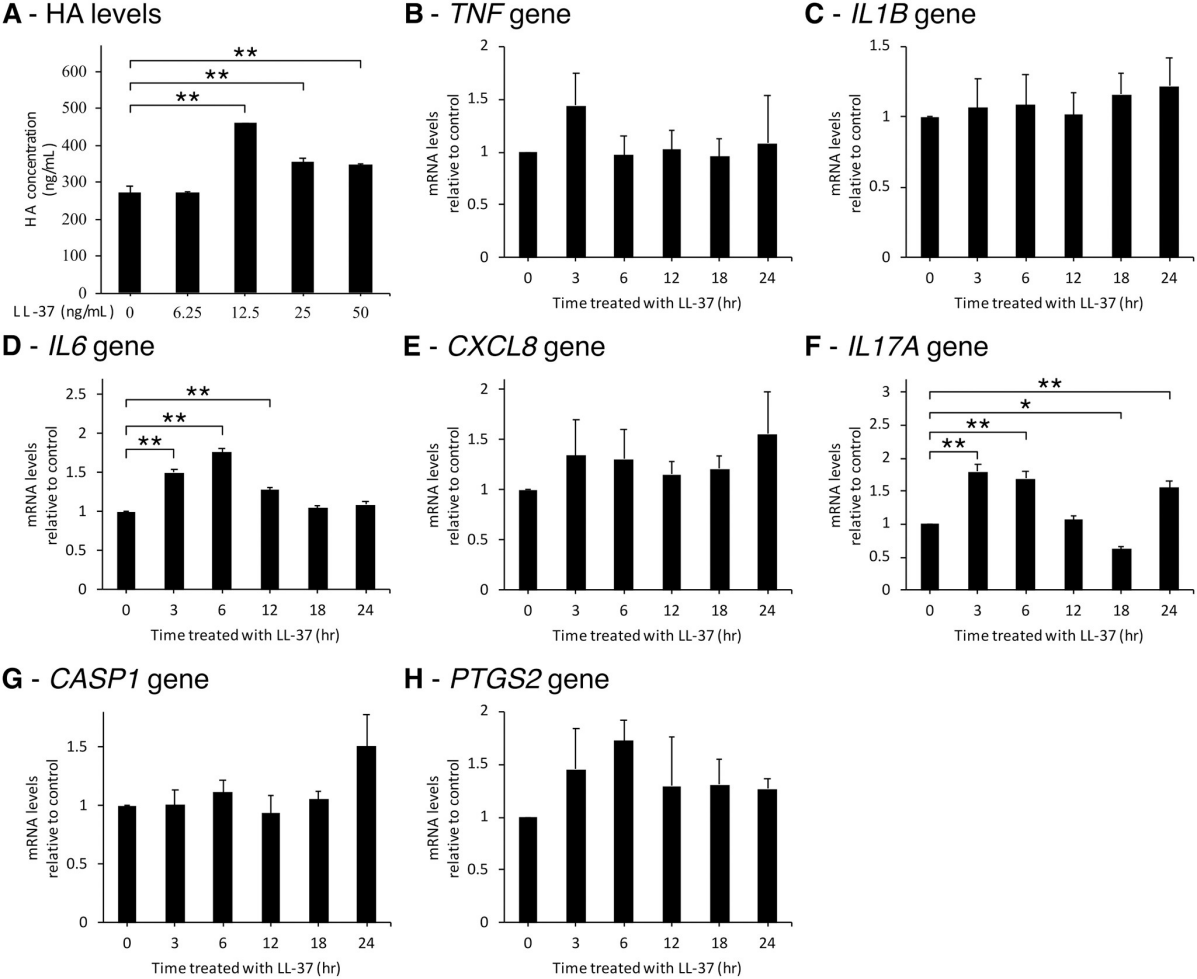
LL-37 induced HA metabolism and inflammatory cytokines and mediators. (A) SW982 cells were treated with varied concentrations of LL-37 for 72 hr. Culture media were collected and measured for HA concentrations. (B-H) SW982 cells were treated with 12.5 ng/mL of LL-37 for varied times. Cells were then collected and measured for the expression of *TNF*, *IL1B*, *IL6*, *CXCL8*, *IL17A*, *CASP1*, and *PTGS2* genes, respectively. The values from triplicate experiments are expressed as the mean ± SD compared to the untreated control. Horizontal brackets indicate significance levels at *p < 0.05 or **p < 0.01.

To find optimal concentration of LL-37 for following experiments and to confirm the effects of LL-37 on HA metabolism, SW982 cells were treated with varied concentrations of LL-37 for 72 hr. The result showed accumulated HA levels in culture media were significantly increased in treatments of LL-37 concentrations from 12.5 to 50 ng/mL and were highest at the concentration of 12.5 ng/mL (Fig 1A). The time-course expressions of inflammatory-related genes were then measured after LL-37 treatment at 12.5 ng/mL. *TNF*, *IL1B*, *CXCL8* or *IL8*, *CASP1* or *ICE*, and *PTGS2* or *COX2* gene expressions were insignificantly increased (Fig 1B, 1C, 1E, 1G and 1H), whereas the expression of *IL6* and *IL17A* genes increased significantly since 3 hr (Fig 1D and 1F).

**Fig 2:**
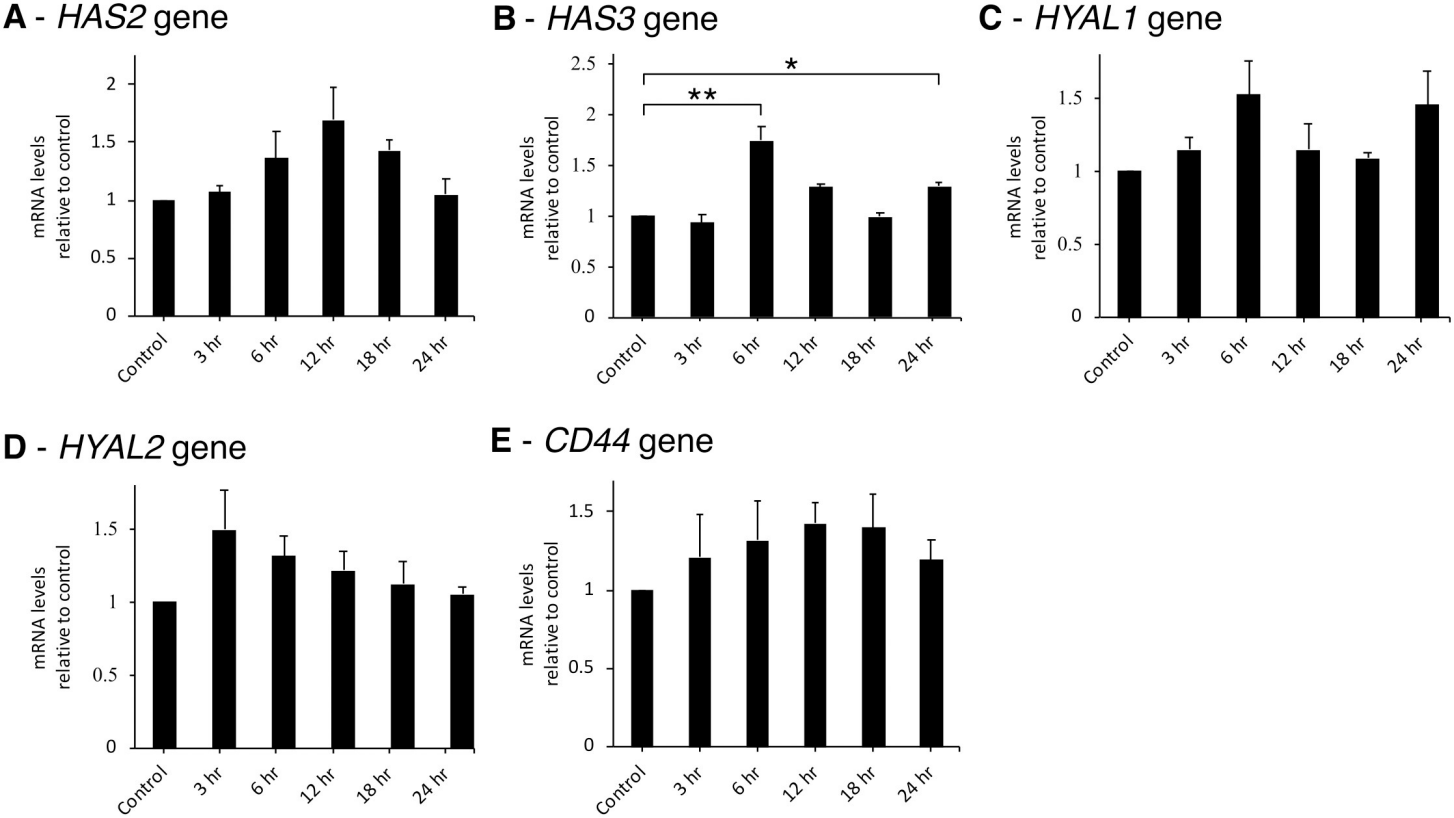
LL-37 induced HA metabolism in SW982 cell line. SW982 cells were treated with 12.5 ng/mL of LL-37 for varied times. Cells were then collected and measured for the expression of *HAS2*, *HAS3*, *HYAL1*, *HYAL2* and *CD44* genes, respectively (A-E). The values from triplicate experiments are expressed as the mean ± SD of fold increases compared to the untreated control. Horizontal brackets indicate significance levels at *p < 0.05 or **p < 0.01.

Time-course experiments investigating the effects of LL-37 at 12.5 ng/mL on the expression of HA metabolism-related genes showed that *HAS2* gene expression increased approximately 60%, reaching a maximum at 12 hr, then declining to basal level (Fig 2A). The expression of *HAS3* and *HYAL1* genes showed similar increasing pattern––highest at 6 hr, then, dropping and increasing again at 24 hr (Fig 2B and 2C), whereas the expression of *HYAL2* gene increased approximately 50% at 3 hr and then declined (Fig 2D). *CD44* expression increased approximately 20% at 6 hr and remained high until 18 hr (Fig 2E).

**Fig 3:**
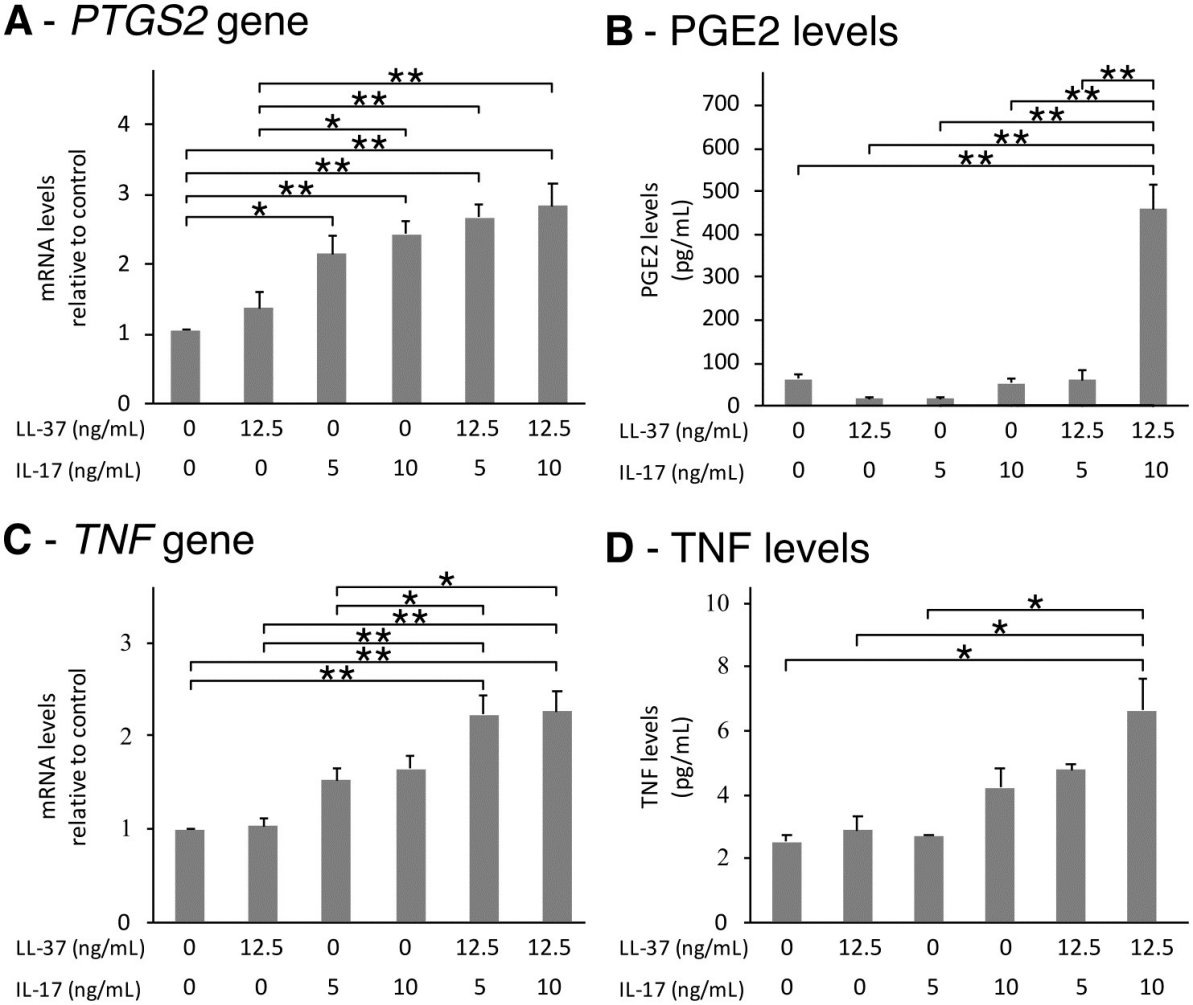
A combination of LL-37 and IL17A enhanced expressions of PTGS2 and TNF. SW982 cells were treated with 12.5 ng/mL of LL-37 and/or 5 or 10 ng/mL of IL17A for 6 hr. Cells were then collected and measured for the expression of *PTGS2* (A) and *TNF* genes (C). The cells were treated for 72 hr before the culture media were collected and measured for PGE2 (B) and TNF level (D). The values from triplicate experiments are expressed as the mean ± SD. Compared to the untreated control and the other treatments, horizontal brackets indicate significance levels at *p < 0.05 or **p < 0.01.

As IL17A was found to express early after cell treatment with LL-37, the combined treatment of LL-37 with IL17A was investigated. Fig 3 shows the effects of combined LL-37 and IL17A on inflammatory cytokine genes and mediators. The *PTGS2* gene was shown to respond to the combinations in a dose-dependent manner (Fig 3A), whereas the levels of PGE2 increased to approximately 5-fold with a combination of 12.5 ng/mL of LL-37 and 10 ng/mL of IL17A (Fig 3B). IL17A significantly induced both *TNF* gene and TNF levels (Fig 3C and 3D).

**Fig 4:**
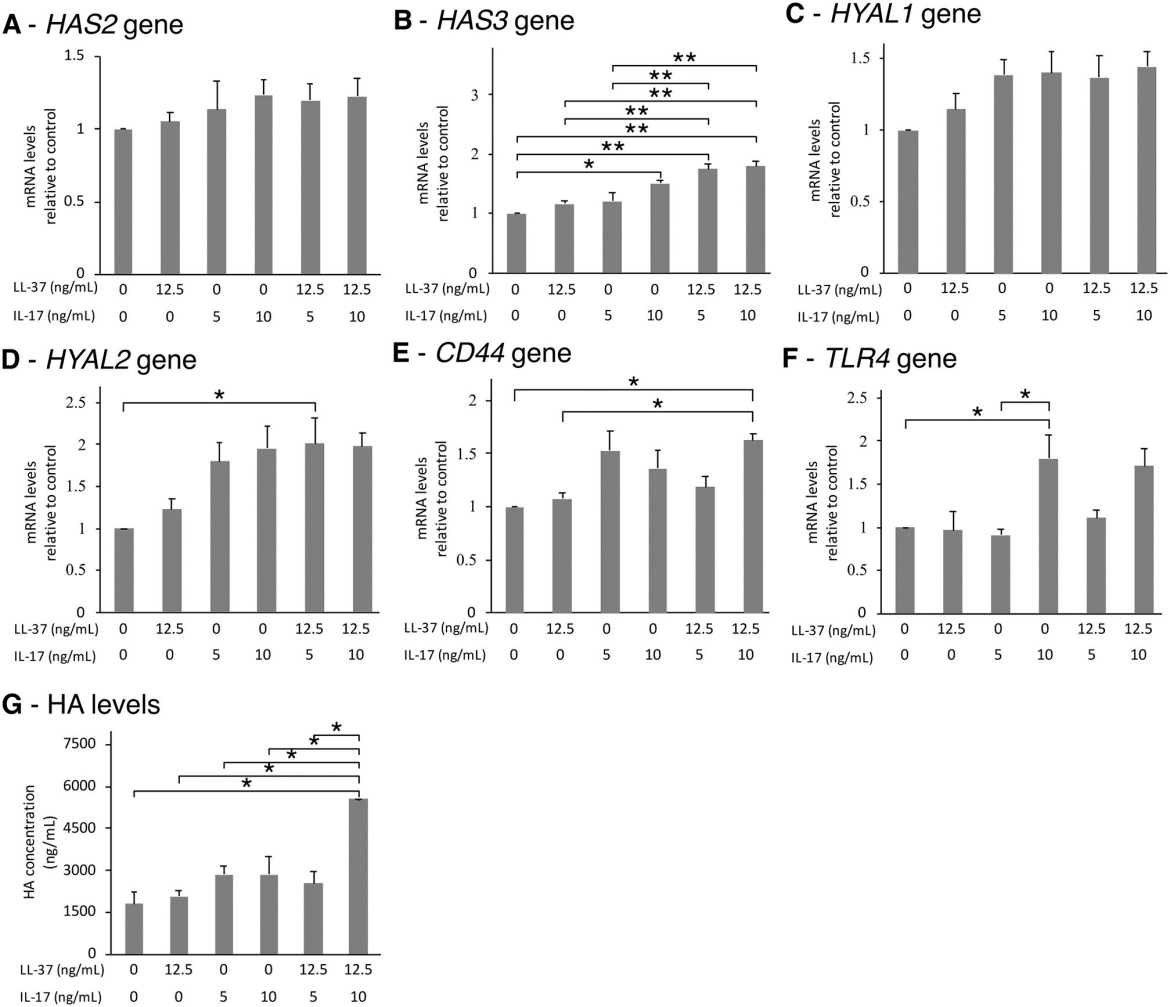
A combination of LL-37 and IL17A induced HA metabolism. SW982 cells were treated with or without 12.5 ng/mL of LL-37 and/or 5 or 10 ng/mL of IL17A for 6 hr. Cells were then collected and measured for the expression of *HAS2*, *HAS3*, *HYAL1*, *HYAL2*, *CD44* and *TLR4* genes, respectively (A-F). SW982 cells were treated with or without 12.5 ng/mL of LL-37 and/or 5 or 10 ng/mL of IL17A for 72 hr before the culture media were collected and measured for HA levels (G). The values from triplicate experiments are expressed as the mean ± SD of fold increases compared to the untreated control. Horizontal brackets indicate significance levels at *p < 0.05 or **p < 0.01.

IL17A combined with LL-37 showed higher gene expression response at 6 hr than LL-37 alone. Although the expression of *HAS2*, *HYAL1*, *HYAL*2, *CD44*, and *TLR4* genes tended to increase following the addition of IL17A, the co-treatments of IL17A and LL-37 did not enhance their activation (Fig 4A and 4C–4F). The effect of LL-37 on the expression of the *HAS3* gene escalated when increasing the concentration of IL17A (Fig 4B). The HA levels in the combined treatment of 12.5 ng/mL of LL-37 and 10 ng/mL of IL17A increased approximately 3-fold compared to control and LL-37 only (Fig 4G).

**Fig 5:**
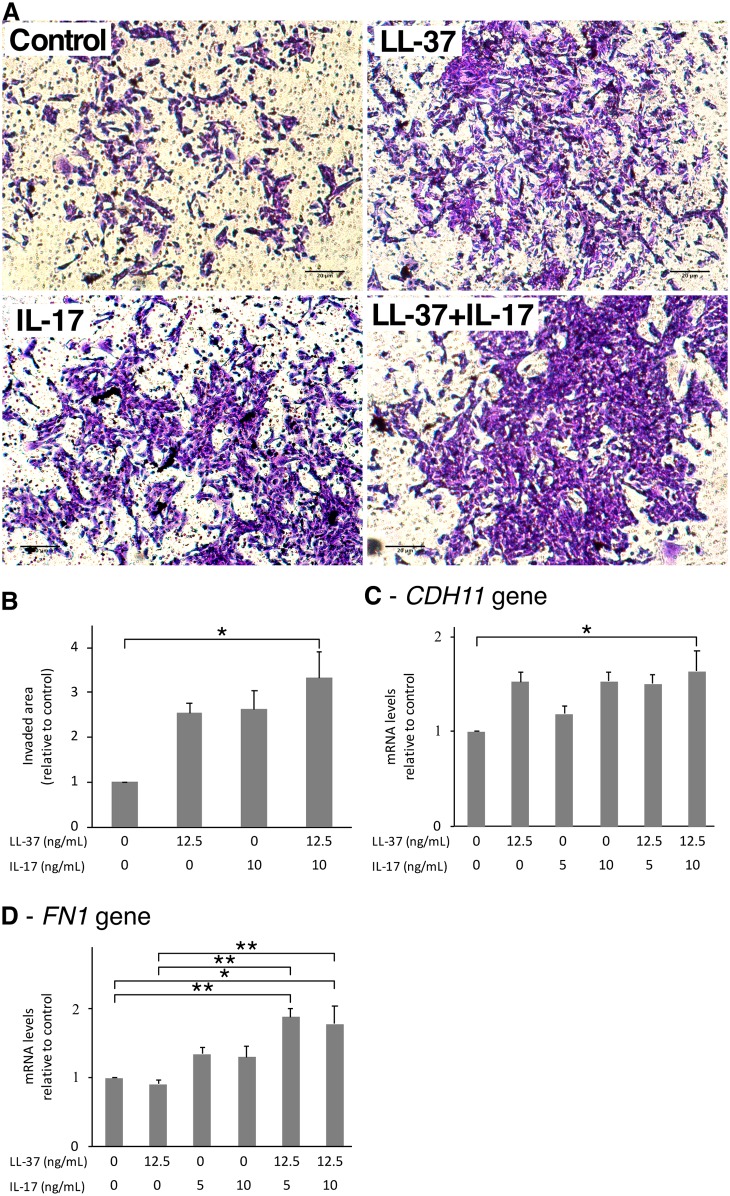
A combination of LL-37 and IL17A induced invasion in SW982 cells. (A) SW982 cells in serum-free L-15 medium containing LL-37 and/or IL17A were seeded into inserts precoated with Geltrex Matrix. L-15 medium supplemented with 20% FBS and treatments was added into the lower chamber. Cells were incubated for 24 hr before being fixed, stained, and photographed. Stained area was measured. Quantitative data were shown in B. SW982 cells were treated with or without 12.5 ng/mL of LL-37 and/or 5 or 10 ng/mL of IL17A for 6 hr. Cells were then collected and measured for the expression of *CDH11* and *FN1* genes (C-D, respectively). The values from triplicate experiments are expressed as the mean ± SD of fold increases compared to the untreated control. Horizontal brackets indicate significance levels at *p < 0.05 or **p < 0.01.

As shown in Fig 5A and 5B, both 12.5 ng/mL of LL-37 and 10 ng/mL of IL17A induced invasion of SW982 cells for 1.5-fold. Moreover, when LL-37 and IL17A were combined, the invasion rate was further increased. Then, the cells were evaluated for invasion-related genes. The *CDH11* gene was induced by both LL-37 and IL17A alone, although when combined they did not increase gene expression (Fig 5C). Although LL-37 alone did not induce *FN1* gene expression, IL17A alone caused an approximately 30% increase. When combined, LL-37 and IL17A increased *FN1* gene expression significantly (Fig 5D).

**Fig 6:**
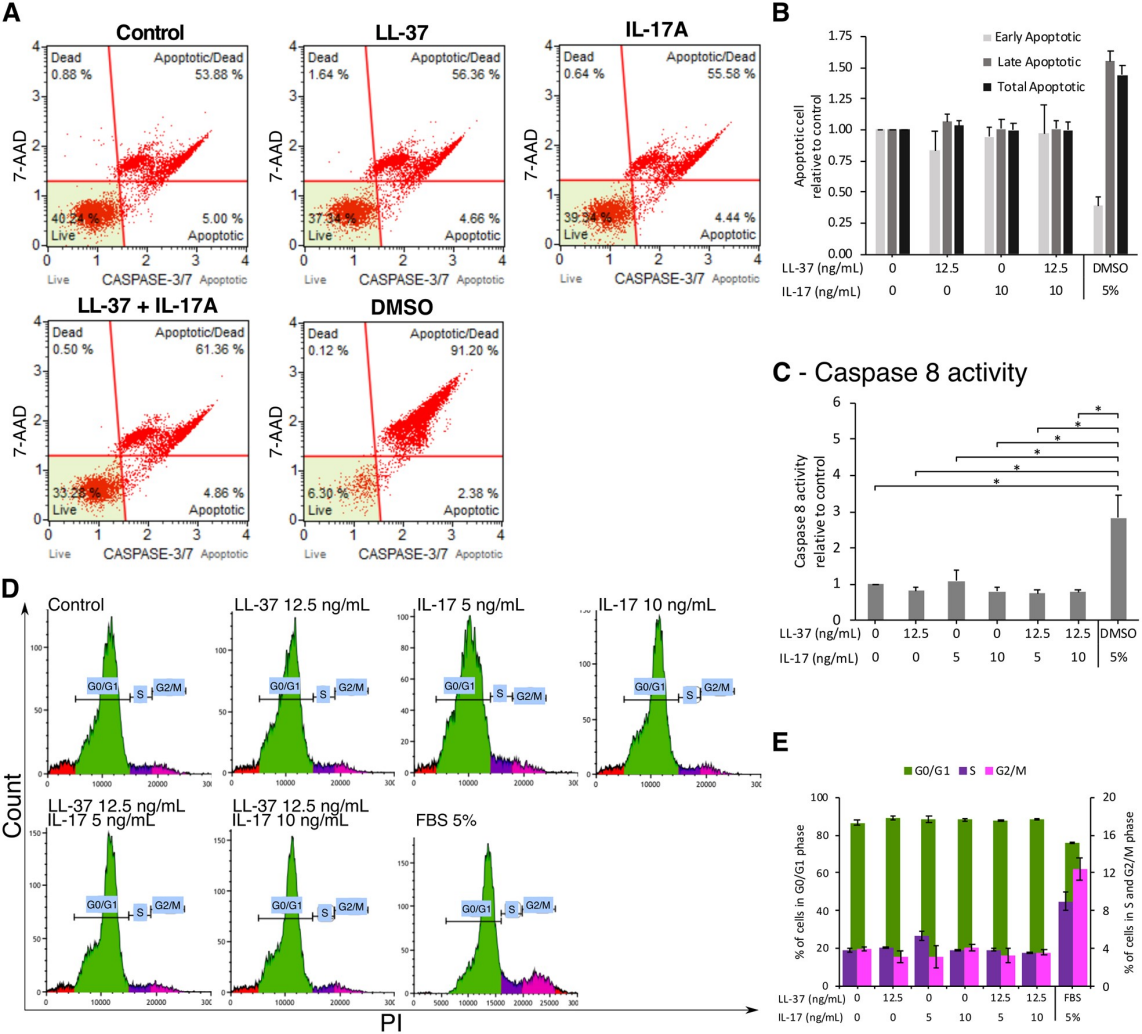
Effects of LL-37 and its combination with IL17A on apoptosis, caspase 8 activity, and cell cycle. SW982 cells were untreated (Ctrl) or treated with 12.5 ng/mL of LL-37 and/or 5 or 10 ng/mL of IL17A for 48 hr before being analyzed. (A and B) Cells were assayed for caspase3/7 activity by a flow cytometer with 5% DMSO as a positive control. (C) Caspase 8 activity was measured by ELISA. (D and E) Cells were stained with PI and checked for cell cycle by a flow cytometer. FBS was used as a positive control. The values from triplicate or duplicate experiments are expressed as the mean ± SD of fold increases compared to the untreated control or percentage. Horizontal brackets indicate significance levels at *p < 0.05.

No significant changes were observed in apoptotic cell numbers among SW982 cells treated with LL-37, IL17A, or their combination (Fig 6A and 6B). Caspase 8 activity was also measured without significant differences observed among treatments (Fig 6C). The cell cycle assay indicated that SW982 cell proliferation was not affected by the treatment (Fig 6D and 6E).

**Fig 7:**
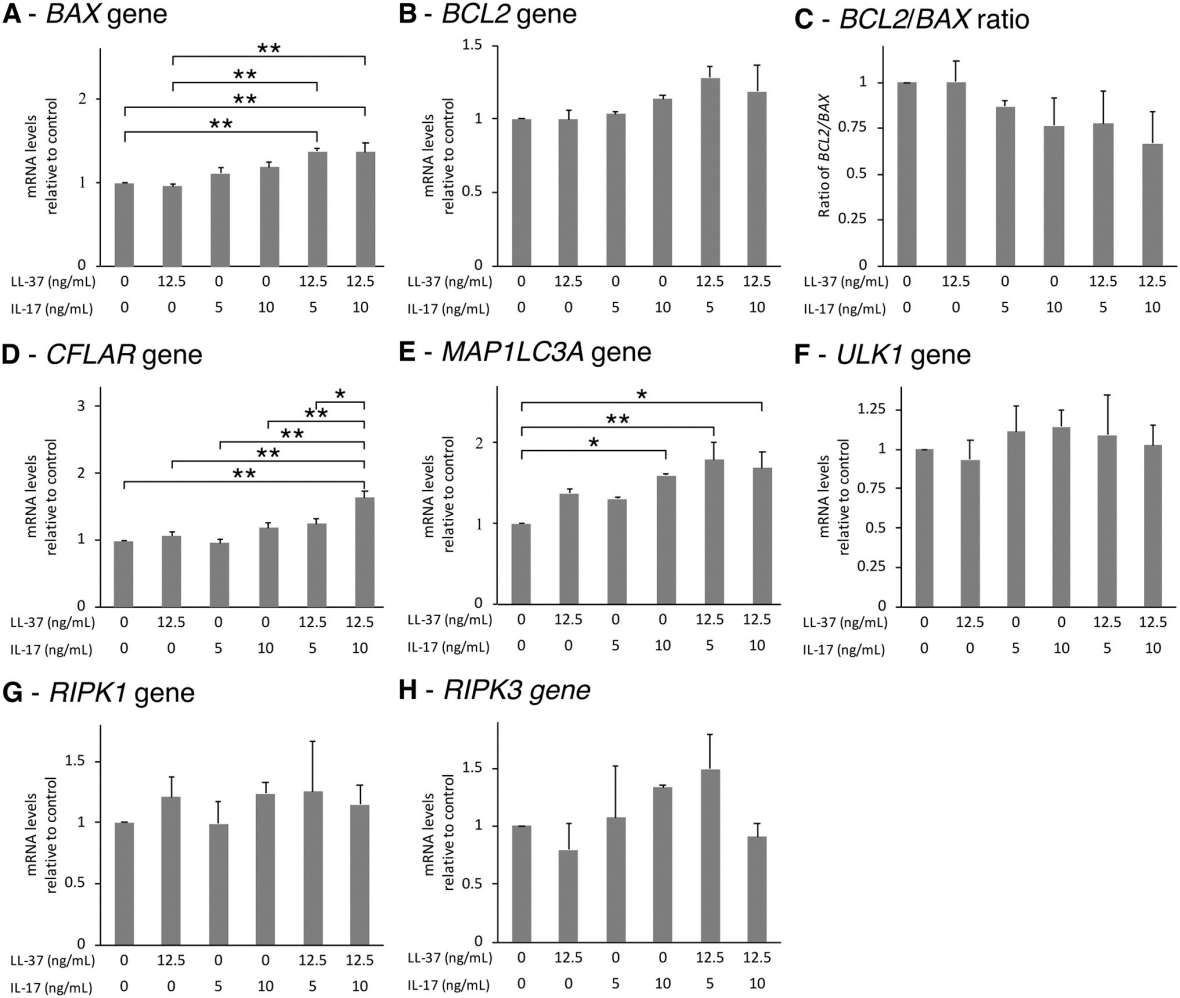
Effects of LL-37 and its combination with IL17A on expressions of genes related to apoptosis, autophagy, and necroptosis. The cells were treated with 12.5 ng/mL of LL-37 and/or 5 or 10 ng/mL of IL17A for 6 hr, then collected and measured for the expression of *BAX*, *BCL2*_,_ *FLIP*, *LC3*, *ULK1*, *RIPK1*, and *RIPK3* genes (A-B and D-H, respectively). Ratios of *BCL2*/*BAX* gene were in C. The values from triplicate experiments are expressed as the mean ± SD of fold increases compared to the untreated control. Horizontal brackets indicate significance levels at *p < 0.05 or **p < 0.01.

Although the expression of *BAX*, *BCL2*, and *CFLAR* or *FLIP* were higher after combination treatment, the ratio of *BCL2*/*BAX*, which represents resistance to apoptosis, was not significantly altered at 6 hr (Fig 7A–7D). The combined treatment of LL-37 and IL17A caused upregulation of *MAP1LC3A* or *LC3*, a marker for autophagy, but not of *ULK1* (Fig 7E and 7F). No significant differences were observed in the expression of *RIPK1* and *RIPK3*, which are necroptosis regulators, following treatment with LL-37 and IL17A (Fig 7G and 7H).

**Fig 8:**
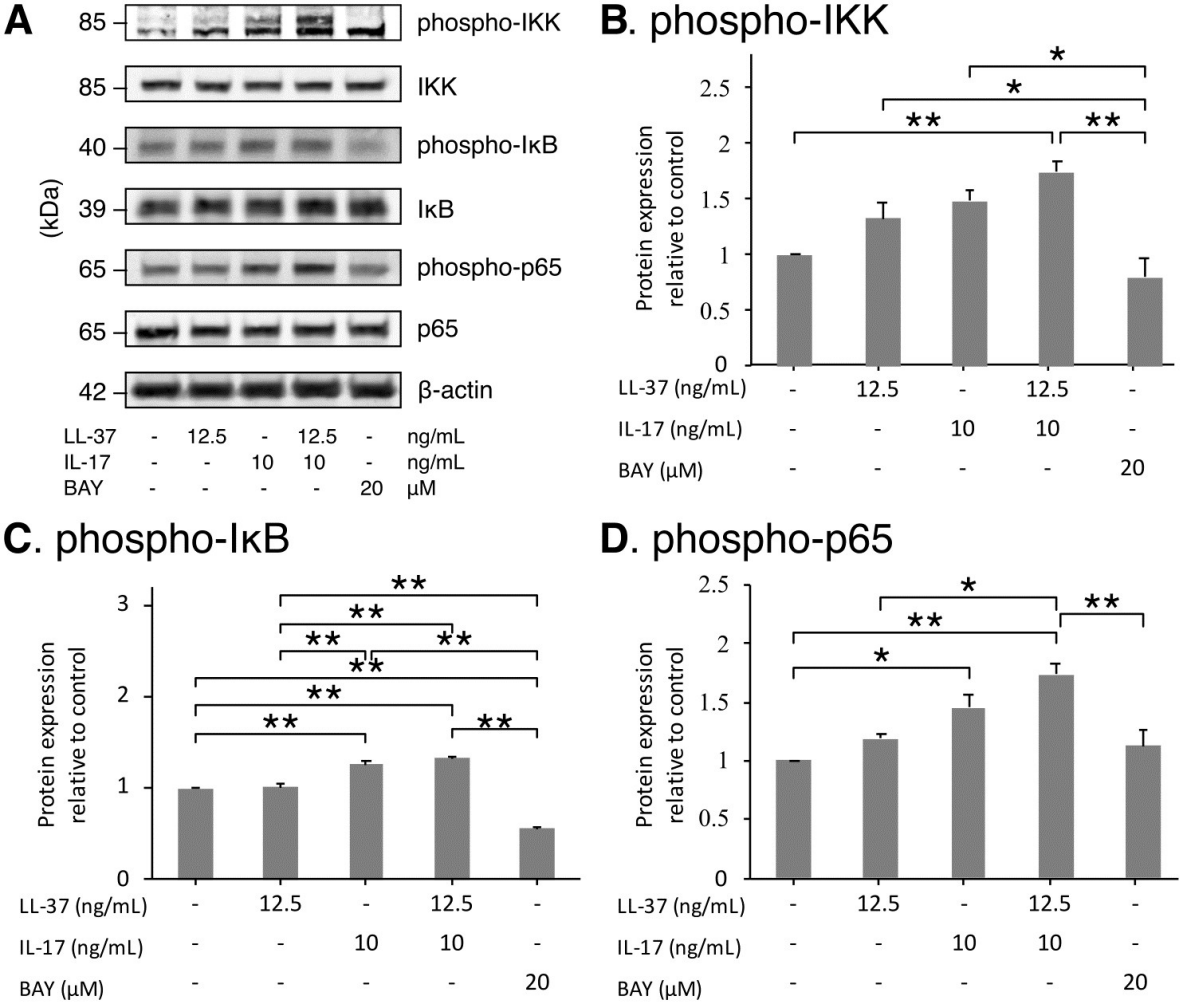
LL-37, together with its combination with IL17A, increased phosphorylated forms of signaling molecules in NF-κB pathway in SW982 cells. The representative of immunoblot against NF-κB signaling molecules is shown in A. B-D are quantitative data of p-IKK, p-IκB and p-p65, respectively. The values from triplicate experiments are expressed as the mean ± SD of fold increases compared to the untreated control. Horizontal brackets indicate significance levels at *p < 0.05 or **p < 0.01. phospho-IKK, phosphorylated IKK. phospho-IκB, phosphorylated IκB. phospho-p65, phosphorylated p65. Band intensity was normalized by respective β-actin intensity and the phosphorylated forms were re-normalized by their total forms.

The inflammation-related NF-κB signaling pathway was examined. LL-37 alone slightly increased the phosphorylation of IKK and p65 but not IκB, whereas IL17A alone significantly boosted the phosphorylation of both IκB and p65 and slightly increased that of IKK. Combined treatment resulted in a noticeable increase in the phosphorylation of IKK and p65. These phosphorylations were suppressed by BAY 11–7082, the NF-κB specific inhibitor (Fig 8).

## Discussion

The present study revealed that LL-37 alone and in combination with IL17A promotes the expression of proinflammatory cytokines and mediators and concomitantly induces the expression of HA-related genes (i.e., *HAS3*, *HYAL1*, *HYAL2*, and *CD44*). These genes have been reported to be associated with inflammatory arthritis and RA pathogenesis [26]. Although cell invasion was strengthened by co-treatment with LL-37 and IL17A, the regulation of apoptosis and cell cycle was not influenced. To our knowledge, this is the first report on the *in vitro* regulatory effects of LL-37, with or without IL17A, on the expression of HA-metabolism-related genes and HA production.

Fibroblast-like synoviocytes (FLS) play an important role in the development of inflammatory arthritis [27]. The use of patient-derived FLS in researches involving the disease is common. However, the FLS has some drawbacks such as the difficulties to collect and establish, the lack of reproducibility due to heterogenous clones and inconsistent results because of non-standardized patient samples. As a model to facilitate the study of inflammatory arthritis pathogenesis, a synovial sarcoma SW982 cell line was chosen due to its convenience, reproducibility and previous research background. Its signaling pathways were investigated and compared with the FLS from patient. It has also been used for drug testing [25, 28, 29]. Nevertheless, the use of only one sarcoma cell line in this study is a big limitation that should be considered because one cell line might not be representative of the key-functioning cells in inflammatory arthritis, including the FLS, where variation among heterogenous groups of cells is generally observed. Therefore, the investigation in FLS is highly required to confirm these phenomenal inductions.

To apply optimal conditions for the study, physiological concentrations of LL-37 were taken into consideration prior to the experiment start. A report with 110 patients measured for their serum LL-37 levels indicated that the concentrations could range from 0.000–26.989 ng/mL [30]. First, we confirmed the effects of varied concentrations of LL-37 on HA production and release and that a significant expression of the proinflammatory cytokines *IL6* and *IL17A* apparently coexisted with *HAS* genes at the early LL-37 activation phase, while other genes, such as *TNF*, *IL8*, and *PTGS2*, also showed increasing trends. This is consistent to the findings by Takahashi et al., who reported that LL-37 could stimulate the expression of IL6 and other inflammatory cytokines [31]. The early LL-37-induced expression of IL6 supports the observation that IL6 mediates the pathophysiology of RA from the acute-phase reaction to the final bone erosion [32]. However, the previously mentioned study reported that levels of serum LL-37 did not correlate with arthritis manifestation [30]. This might be due to the tissue-specific patterns of LL-37 actions being reported in the last decade [33–38], making the study of LL-37 regulatory effects or interactions in specific microenvironment worth performing.

IL17A is another proinflammatory cytokine found to respond against LL-37 activation at the early time of 3 hr. The involvement of this cytokine in the development of RA is highly convincing. Jovanovic et al. reported that IL17A was detected in the synovial fluid of RA patients, where it upregulated MMP-9 in a dose- and time-dependent manner, as well as PTGS2 [39]. On the other hand, it was not detected in OA patients or in control FLS. Adamopoulos et al. reported that IL17A activated the activator receptor for NF-κB receptor on human osteoclast precursors *in vitro* [40]. This suggests that IL17A is involved in RA pathogenesis, specifically in the degradation of the surrounding cartilage. IL17A and IL6 co-expression at an early phase of LL-37 induction suggests that, similar to IL6, IL17A is involved in several systemic manifestations of RA pathogenesis. Therefore, further investigation into the effects of combined IL6 and IL17A is of interest.

In FLS, *HAS1* gene expression is very low or undetectable, whereas *HAS2* and *HAS3* function constitutively [41]. A high expression of *HAS3* was observed in the synovium of RA patients as well as in other inflammatory arthritis diseases [26], but rarely in osteoarthritis [42]. A previous study on SW982 culture by Viriyakhasem et al. [25] supports the existence of an association between HA metabolism and inflammation, as it found that lipopolysaccharides simultaneously induced the expression of *IL1B*, toll-like receptor 4 (*TLR4*), IL-1β-converting enzyme (*ICE or CASP1*), *HAS2*, *HAS3*, and *CD44* whereas the expression of *HAS1* gene remained undetectable. The same study suggested that the SW982 cell line could be used as an effective tool to study these two pathways in parallel.

The present study demonstrated the time-course effects of LL-37 on the expression of both HA-metabolism-related genes and proinflammatory cytokines and mediators. The expression of *HAS3*, *HYAL1*, *HYAL2*, and *CD44* genes showed a similar significant upregulation at 3–6 hr, although the subsequent expression pattern differed among genes; the first two showed a sharp decrease, while *CD44* maintained a high expression until 18 hr. *HAS2* mRNA, on the other hand, showed an increasing trend, peaking at 12 hr, then, dropping to the basal level at 24 hr. These variations were confirmed by the significant increase of HA release and accumulation in the culture media. HYAL1 and HYAL2 are the major mammalian hyaluronidases in somatic tissues, and they act in concert to degrade high molecular weight hyaluronan into tetra-saccharide molecules. Although the method used in this study is unable to distinguish the size of accumulated HA, the co-expression of *HAS* genes and hyaluronidases in response to LL-37 activation suggests that this may cause the degradation of HA into smaller fragments, exerting a proinflammatory action [43]. Therefore, the size of accumulated HA could be a subject of further investigation.

According to its early response to LL-37 activation, we proofed that an addition of IL17A amplified the activating effects of LL-37 on the co-expression of *HAS* genes, together with the expression of proinflammatory cytokines involved in RA pathogenesis. The combined treatment with LL-37 and IL17A significantly enhanced the stimulating effects on *HAS3* expression and the accumulated level of HA level, while *HYAL2* and *HYAL1* genes seemed to respond to IL17A rather than LL-37. These may lead to a massive generation of small HA fragments, which probably results in a multiplying magnitude of response to prolong the cascade of proinflammatory cytokines expression.

Moreover, the combination of LL-37 and IL17A enhanced the gene expression of *PTGS2* and *TNF*, while increasing the levels of PGE2 and TNF for approximately two- and three-fold, respectively. It is noteworthy that LL-37 alone did not induce TNF expression significantly, although in combination with IL17A, it boosted its expression higher than IL17A alone. TNF plays an important part in chronic inflammation and induces inflammatory arthritis development. It also promotes the release of several inflammatory mediators from FLS and other cells infiltrating the joints [32]. A similar effect was found for PTGS2, except that its expression was induced by LL-37 alone, and to a higher degree with combined treatment. This finding is consistent with a previous report by Chotjumlong et al., who found that LL-37 upregulated the expression of PTGS2 and the synthesis of PGE2 in human gingival fibroblasts [21]. It has been suggested that PGE2 plays an important role in chronic inflammatory diseases and cancer [44]. However, there are contradicting reports on how LL-37 affects proinflammatory cytokines as well. It is found that exogenous LL-37 decreased TNF and IL17A expression while inducing anti-inflammatory IL-10 and TGF-β production in dendritic cells in allergy and inhibit LMW-hyaluronan-induced cytokine release in skin fibroblast [35, 36]. These contradicting results might be due to different cell types, different disease conditions, or different LL-37-interacting molecules.

Additionally, our results suggest that the combination of LL-37 with IL17A may relate to the chronic inflammatory processes by enhancing the production of proinflammatory cytokines, especially TNF, together with the proinflammatory mediator, PGE2. These may contribute for a greater production of HA as reported previously by Honda et al., who found that PGE2 induced HA synthesis through cAMP-mediated protein kinase signal transduction [45]. Another molecule that may link LL-37 to the production of HA is its cell-surface receptor, CD44. CD44 is a widely distributed transmembrane glycoprotein involved in the regulation of cAMP-mediated signaling [16]. The mechanism underlying the effects of combined LL-37 and IL17A on the expression of HA-related genes and the amount of HA produced, should be investigated further to clarify whether the cAMP-mediated signaling is directly involved, or results from the activation by the PGE2 present in the culture media. On the latter aspect, Honda et al. found that adding 50 ng/mL of prostaglandin for 6 hr could significantly induce HAS activity in rabbit pericardial cells [45], while the amount of PGE2 measured after incubation with LL-37 and IL17A for 72 hr was approximately 400 pg/mL. Although this is quite a low amount compared with the previous report, the possibility that PGE2 activation might involve HA-related gene expression and HA production still should be examined considering the longer exposure time in our case and fluctuation of PGE2 itself.

TLR4, another important receptor for HA, showed response to IL17A at higher concentration, but not to LL-37. This may suggest that it responds to high level of inflammation [25] that in turn results from (i) the higher production of LMWHA due to upregulation of *HAS3*, *HYAL1* and *HYAL2* genes and (ii) the upregulation of inflammatory cytokines by IL17A. However, *TLR4* expression showed selective inducibility as LL-37-induced inflammation did not upregulate *TLR4* expression. TLR4 binding with LMWHA was also found to induce inflammatory cytokine production through the NF-κB pathway or type-1 interferons [17]. Together, this LMWHA-TLR4-IL17A cycle would help with the continuity of inflammatory cycle.

To investigate the pathway linking LL-37 to the downstream changes, we looked into IKK, IκB, and p65, the main components of the NF-κB pathway that usually activate the expression of proinflammatory cytokines and mediators, leading to the onset of inflammation [46]. Our results showed that LL-37 slightly increased the phosphorylation of both IKK and p65, which was clearly enhanced by the co-treatment with IL17A, whereas IL17A was found to be a better inducer for IκB phosphorylation. These results suggest that enhancement of NF-κB molecules phosphorylation by the combination of LL-37 and IL17A may be directly associated with a dramatic increase in the expression and synthesis of TNF and PGE2. In addition, other approaches such as flow cytometry or reporter assay are suggested for the confirmation of the results. It is also noteworthy that generally primary cells and cell lines respond to inflammatory cytokines and host-defense peptides differently especially in cell signaling regulated by the phosphorylation of protein. This, thus, emphasizes the necessity of further investigation in FLS.

In RA pathogenesis, the increase of cell invasion, proliferation, and resistance to apoptosis of FLS bolsters disease progression [27, 47, 48]. The present study revealed that either LL-37 or IL17A induced the invasion of SW982 cells, with their combination increasing the invasion rate further, consistent with the enhancement of the fibronectin gene, *FN1*, expression. The gene of cadherin11, *CDH11*, was found to be upregulated by both LL-37 and IL17A alone, but not by their combination, suggesting that the combined LL-37 and IL17A may provide an additional selective cascade to enhance cell invasion via upregulation of fibronectin expression. Immunoblotting showed that the combined LL-37 and IL17A increase the phosphorylation of the NF-κB signaling pathway. It has been reported previously that the high expression of fibronectin in colorectal cancer is associated with cell proliferation and migration via the NF-κB/p53-apoptosis signaling pathway [49]. However, our results revealed that the increase of fibronectin expression by the combined LL-37 and IL17A in the SW982 cell line neither coexisted with cell cycle change nor affected the apoptotic process, suggesting a unique condition induced by the combined LL-37 and IL17A, which is worth further investigating.

The affected FLS show apoptosis-resistance, leading to hyperplasia or enlarged pannus formation. It has previously been reported that LL-37 induces apoptosis in Jurkat T leukemia cells [50], while IL17A either promotes or induces apoptosis in different cell types [51, 52]. In the present study, neither LL-37 nor its combination with IL17A altered the apoptotic process of SW982 cells, as confirmed by flow cytometry of caspase3/7 activity and by the expression ratio of the apoptotic genes, *BCL2/BAX*. The *CFLAR* or *FLIP* gene, which is designated as a master anti-apoptotic regulator [53], was significantly upregulated after the combined treatment of LL-37 and IL17A, consistent with the unchanged levels of caspase 8. Although this result suggests the onset of apoptotic resistance under this treatment, the unchanged ratio of *BCL2*/*BAX* genes and unaltered cell cycle within 48 hr of treatment was observed. The ability to induce apoptotic resistance and cell proliferation by the combined LL-37 and IL17A needs further investigation.

CFLAR or FLIP is also involved in necroptosis and autophagy. It is known to interfere with the localization of Atg13 to the autophagosome, thus, inhibiting LC3 maturation [54]. In the present study, upon measuring *LC3* gene expression we found that the combined treatment with LL-37 and IL17A significantly increased it, in response to the depleting amount of functioning LC3-II caused by maturation interference by CFLAR. However, measuring autophagic activity requires the use of western blotting of LC3 or LC3 dot count, thus needed further investigation. Next, we looked into the gene expression of two necroptotic regulators, *RIPK1* and *RIPK3*. The results suggest that neither LL-37 nor its combination with IL17A affected necroptosis.

As previously mentioned, inflammatory arthritis patients have higher serum HA, synovial HA or synovial LL-37 level [7, 20, 24, 42], and our results showed that, in a microenvironment of SW982 cell, these individual physiological conditions could coincide and have regulating effects on one another. Thus, we propose that these phenomena might occur in the patient-derived FLS as well and we strongly encourage expanding these valuable finding by an in-depth investigation on primary FLS.

In conclusion, we demonstrated that LL-37 rapidly induced inflammation-related phenomena, including upregulated expression of proinflammatory cytokine genes and co-expression of HA-metabolism-related genes. *IL17A*, which was early activated by LL-37, in combination with LL-37 enhanced those phenomena, together with cell invasion but not cell cycle nor cell apoptosis. These results suggest that, under chronic LL-37-inducing conditions, LL-37 may synchronize with the downstream proinflammatory cytokines, contributing to the co-operative enhancement mechanisms associated with inflammation, especially in inflammatory arthritis pathogenesis.

## Supporting information

S1 Table List of primers. (DOCX)

S1 Fig Blot images of Fig 8. (PDF)

S1 Data Raw quantitative data of the results. (XLS)
