# Supplementary material for: LL-37 alone and in combination with IL17A enhances proinflammatory cytokine expression in parallel with hyaluronan metabolism in human synovial sarcoma cell line SW982—A step toward understanding the development of inflammatory arthritis
Source: PLoS One. 2019 Jul 1;14(7):e0218736. doi: 10.1371/journal.pone.0218736 (PMC6602187; doi:10.1371/journal.pone.0218736)
Supplement: S1 Table — (DOCX) [file pone.0218736.s001.docx]

**S1 Table. Primer list**

­­

| Genes | Forward (5' to 3') | Reverse (5' to 3') |
| --- | --- | --- |
| BAX | AGCAGATCATGAAGACAGGGG | ACACTCGCTCAGCTTCTTGG |
| BCL2 | TGTGTGTGGAGAGCGTCAA | GACAGCCAGGAGAAATCAAACAG |
| CD44 | TCATTACCCACACACGAAGGA | TGGAATGTGTCTTGGTCTCCTG |
| COX2 | GTTCCACCCGCAGTACAGAA | AGGGCTTCAGCATAAAGCGT |
| Fibronectin | CTGGCCGAAAATACATTGTAAA | CCACAGTCGGGTCAGGAG |
| FLIP | TTTTGTGCCGGGATGTTGCT | GAGCAGGTCAAATCGCCTCA |
| GAPDH | GAAGGTGAAGGTCGGAGTC | GAAGATGGTGATGGGATTTC |
| HAS1 | CCTGCATCAGCGGTCCTCTA | GCCGGTCATCCCCAAAAG |
| HAS2 | ATTGGAACCACACTCTTTGGAG | TGAGGTGTGATGCCAAAAAGG |
| HAS3 | TTATCAAGGCCACCTACGCC | ATGGTTTTTCGGCCAGAGGT |
| Hyal1 | ATGGCTTCCCTGACTGCTAC | GGTCATTTTGGGCACGGATG |
| Hyal2 | GACCGCATAGTCAAACAGGC | ATAACGCAGTGTCTCCAGCA |
| IL17A | TTGGTGTCACTGCTACTGCT | TTGGGCATCCTGGATTTCGT |
| IL1β | CCTGTCCTGCGTGTTGAAAGA | GGGAACTGGGCAGACTCAAA |
| IL6 | GGTACATCCTCGACGGCATCT | GTGCCTCTTTGCTGCTTTCAC |
| IL8 | ACACTGCGCCAACACAGAAA | CAACCCTCTGCACCCAGTTT |
| LC3II | GAGAAGCAGCTTCCTGTTCTGG | GTGTCCGTTCACCAACAGGAAG |
| LL37 | CCAGGGGCTCCTTTGACATC | TCCTGGGTACAAGATTCCGC |
| TLR4 | CTGCAATGGATCAAGGACCA | TTATCTGAAGGTGTTGCACATTCC |
| TNFα | CCCCAGGGACCTCTCTCTAATC | GGTTTGCTACAACATGGGCTACA |
| ULK1 | GTCCAGCTTTGACAGTCAGT | TCCCAGGACTCAGGATTCCA |
